# Relationship between oral health impacts and personality profiles among orthodontic patients treated with Invisalign clear aligners

**DOI:** 10.1038/s41598-020-77470-8

**Published:** 2020-11-24

**Authors:** Abdullah A. Al Nazeh, Ibrahim Alshahrani, Serene A. Badran, Salem Almoammar, Abdulaziz Alshahrani, Bashar A. Almomani, Mahmoud K. AL-Omiri

**Affiliations:** 1 Department of Pediatric Dentistry and Orthodontics Sciences, College of Dentistry, King Khalid University, Asir–Abha, Saudi Arabia; 2 Department of Paediatric Dentistry, Orthodontics and Preventive Dentistry, School of Dentistry, University of Jordan, Queen Rania Street, Amman, 11942 Jordan; 3 Division of Orthodontics and Oral Maxillofacial Surgery, Department of Dentistry, King Hussein Medical Center, Royal Medical Services, Amman, Jordan; 4 Department of Prosthodontics, School of Dentistry, University of Jordan, Queen Rania Street, Amman, 11942 Jordan; 5 Department of Prosthodontics, The City of London Dental School, Canada Water, Lower Road, London, UK

**Keywords:** Orthodontics, Dentistry, Oral-health-related quality of life

## Abstract

This within subject clinical experiment assessed oral health impacts before and after Invisalign orthodontic treatment and their relationships with personality characteristics. 50 patients (26 females and 24 males; mean age = 27.62 ± 8.25 years, SE = 1.17, 95% CI = 24.71–29.89 years) were assessed before and after treatment with Invisalign orthodontic treatment. Treatment clinical success was evaluated according defined clinical guidelines. Oral health impacts before and after Invisalign orthodontic treatment were measured via the Oral Health Impact Profile (OHIP). Personality features were measured via the NEO Five-Factor Inventory (NEO-FFI). Probability of α = .05 was utilized to identify significant findings. Females scored less OHIP scores after treatment (had less negative impacts) in comparison to baseline OHIP scores (t = 3.782, df = 25, *P* = .001, 95% CI of mean difference = 2.750–9.327). Among males, openness scores (R^2^ = .911, B = 5.235, 95% CI for B = 0.062–10.407, t = 2.601, *P* = .048) were able to predict OHIP scores before treatment; meanwhile, extraversion (R^2^ = .959, B = − 8.224, 95% CI for B = − 14.605–1.843, t = − 3.313, *P* = .021), openness (R^2^ = .959, B = 21.795, 95% CI for B = 10.737–32.853, t = 5.067, *P* = .004), and conscientiousness (R^2^ = .959, B = 10.293, 95% CI for B = 4.796–15.790, t = 4.813, *P* = .005) scores were useful to predict OHIP scores after treatment (R^2^ = .959, *P* < .05). NEO-FFI scores were not useful to predict OHIP scores before or after treatment among females (*P* > .05). These findings demonstrate that oral health impacts of Invisalign orthodontic treatment and personality profiles contribution to oral health impacts were different between genders.

## Introduction

Oral health related quality of life, oral health impacts and personality features were associated among participants with various oral conditions and dental treatments including orthodontic treatment^1–8^. Nevertheless, this association was not identified by other researchers^9–11^. This variance, along with adopting unpredictable techniques to measure personality and dental impacts and satisfaction, makes it necessary to carry out more evidence based inquiries to provide decisive conclusions in this regard. Furthermore, this should be achieved by suitable measures and questionnaires that possess adequate validity and reliability^1–9^.

Invisalign is a well known clear aligner that marked contemporary orthodontics. However, clear orthodontic aligners still have limitations, and were found to be less accurate and less effective than fixed orthodontics^12^. Nevertheless, previous studies showed some advantages of Invisalign over fixed orthodontic appliances including better oral hygiene, patient comfort, esthetics, periodontal health, and reduced pain levels, treatment time and clinical time^12–16^.

Although oral health related quality of life was improved after fixed orthodontic treatment^17^, Invisalign was associated with better oral health related quality of life in comparison to fixed orthodontics^18–22^. This is especially evident considering better eating and chewing as well as less ulcerations and pain^20,21^. However, Invisalign was found to be associated with inferior oral health related quality of life regarding speech in comparison to fixed orthodontic appliances^21^.

Nonetheless, patients’ approval, perception, and satisfaction with oral health and treatment might not be secured even if they receive adequate dental treatment^1–8^. Personality attributes might underlie and explain such observation^1–8^.

Some relationships between personality, oral health impacts, and dental satisfaction were previously established following other treatments or various dental managements and situations^1–11^. Nevertheless, studies on oral health impacts following Invisalign orthodontic treatment are scarce. In addition, no previous investigations evaluated the correlation between personality attributes and oral health impacts of Invisalign orthodontic treatment. Taking this into consideration, it is worth investigating these aspects in an attempt to provide more evidence concerning best treatment in this regard as well as to uncover the relationships between personality and oral health impacts that associate Invisalign orthodontic treatment.

The aim of this inquiry was to appraise the correlations between participants’ personality attributes and oral health impacts before and after Invisalign orthodontic treatment (Invisalign, Align Technology, Inc., Santa Clara, CA, USA). The null hypothesis for this inquiry was specified as no correlations are present between personality attributes and oral health impacts before and after Invisalign orthodontic treatment.

## Methods

### Study design, population and instruments

The present within subject longitudinal observational clinical study was performed in ethical harmony with Helsinki Declaration (9th version, 2013). It was ethically authorized by the Institutional Review Board, (Research Ethics Committee, King Khalid University, Saudi Arabia; Reference number: IRB/KKUCOD/012). Participants were provided with thorough explanation of different aspects of this investigation. Informed consent was obtained from all participants for the experiment.

Fifty participants (26 females and 24 males) who attended the orthodontic clinics at the College of Dentistry (King Khalid University, Saudi Arabia), and were planned to be managed with Invisalign orthodontic treatment to treat their malocclusion, joined this inquiry. This investigation was performed between May 2018 and December 2019. Simple randomization method with gender stratification was used to select participants from the waiting list by assigning computer produced numbers to them.

The participants were eligible to join the study if they were above 18 years old, and did not receive previous orthodontic treatment. They should have received no previous surgical, prosthodontic or implant treatment. Also, they should have no local problems including bone lesions and teeth problems like periodontitis, caries, tooth fractures, tooth wear, or endodontic problems. In addition, they should suffer no treatment failure or clinical problems during this investigation. Participants should not be on medication or endure medical issues and problems including immune disorders, bone disease, bleeding disorders, cardiovascular disease, gastrointestinal problems, liver disease, renal problems, or endocrine disease. They should also have no mental illness that interferes with their comprehension or ability to score the tests.

Nevertheless, participants younger than 18 years old, pregnant women, participants experienced any of the above medical problems, and alcoholics were excluded and not eligible to join this inquiry. Also, participants who experienced treatment failures during this inquiry or received previous orthodontic, prosthodontic, implant, or surgical treatment at the jaws were excluded from the study. In addition, participants were excluded if they experienced active local bone disease or dental problems that affect the dentition.

Following inclusion in this investigation, detailed appraisal of the participants’ medical health, dental history, oral complaints, and individual records including age, gender, level of education, marital status, and smoking was carried out. Then, two orthodontists performed detailed appraisal of clinical status, dental factors and radiographic findings for each participant following earlier recommendations^2,7,9,23–25^. The clinical assessment included orthodontic evaluation of the skeletal pattern, incisor classification, canine classification, molar classification, degree of overbite, degree of overjet, presence of cross bite, presence of scissor bite, the need for extraction, and presence of tooth crowding/spacing.

The participants were clinically examined on a dental unit utilizing a dental mirror (15/16 inch; Hahnenkratt, Königsbach-Stein, Germany) and a single ended explorer (0700-9; ASA Dental, Bozzano, Italy). The radiographic assessment was performed utilizing periapical, panoramic (OPG), and lateral cephalometric radiographs following previous recommendations^2,7,9,23–25^. Intraoral and extraoral photographs were obtained using a Canon camera with Canon macro-lens and flash ring (Canon EOS 60D, Canon Inc., Japan).

Before starting the treatment, the participants’ personality attributes and characteristics were measured via Neuroticism Extraversion Openness Five Factor Inventory (NEO-FFI)^26^. In addition, oral health impacts were measured for each participant via Oral Health Impact Profile (OHIP)^27^. The purpose, contents, and method of scoring each test was explained to each participant. They were informed that the investigators will be available to provide them with any required explanation or information in this regard.

The OHIP has 14 items that are scored on a 5 point Likert scale (response from never to very often). It measures self-reported oral health impacts including dysfunction, discomfort, and disability^27^. It is valid, reliable, short and easy to score^27,28^, therefore, it was used to assess oral health impacts during this inquiry.

Meanwhile, the NEO-FFI inventory has 60 questions that are scored on a 5 point Likert scale (response from strongly agree to strongly disagree). It assesses the 5 major personality aspects: neuroticism, extraversion, openness, agreeableness and conscientiousness. It allows complete assessment of personality characteristics and can be scored quickly and easily; while at the same time, it is sensitive, reliable, valid, and accurate^1–9,26,29^. Besides, some former studies in the field of personality and dental satisfaction endured the pitfall of employing tests that did not evaluate the main five aspects of personality and problems concerning their reliability, validity, sensitivity, and suitability to use^1–9,26,29^. Therefore, the NEO-FFI inventory was used to assess personality attributes during this inquiry.

After scoring the NEO-FFI, OHIP, and VAS measures; the participants’ treatment with Invisalign was initiated following careful assessment of each patient according to previous recommendations and manufacturer’s instructions. Intraoral scans were taken in the clinic using iTero Element scanner (Align Technology, Inc., Santa Clara, CA, USA), and were sent to the manufacturer (Align Technology, Inc., Santa Clara, CA, USA). Tooth movements were planned using ClinCheck software (Align Technology, Inc., Santa Clara, CA, USA). The digital plan was then finalized, double checked, and approved by the investigator and the participants. Then, the Invisalign trays (Align Technology, Inc., Santa Clara, CA, USA) were fabricated by the manufacturer under controlled conditions within special company labs using robots and lasers to mold and produce the trays via three dimensional printing. The trays were then delivered to the investigator. The Invisalign trays were then checked by the investigator, and the first tray was fitted in the patients’ mouth. SmartForce attachments (Align Technology, Inc., Santa Clara, CA, USA) were used for participants to secure retention of the Invisalign trays. The participant was given instructions for using, fitting and cleaning the Invisalign trays, and was then dismissed and was followed up monthly to monitor treatment progress as well as to check for any patient complaints or any problems with the trays.

Three months after finishing the treatment, two investigators (consultant orthodontists) performed detailed clinical and radiographic assessment to evaluate treatment success according to previous recommendations^2,7,9,23–25,30^. The success of treatment was assessed by observing the keys for normal occlusion and the ability to obtain correct molar relationship, correct crown angulation and inclination, no rotation, no spaces, flat occlusal table, and adequate root angulation^2,7,9,23–25,30^. For this purpose tooth alignment, tooth buccolingual inclination, marginal ridges position, overjet, occlusal relations, occlusal contacts, interproximal contacts, and root angulation were observed following previous recommendations^2,7,9,23–25,30^. The success of treatment was also judged based on accuracy of movements/planned movements via ClinCheck of Invisalign, changes in alignment, changes in occlusion, and completion of planned treatment.

It was planned to exclude participants who had appliances that did not correct the dental problem, were associated with treatment failure, or were associated with adverse incidents and unwanted outcomes. Also, participants would have been excluded if their appliances suffered poor retention, fractured, or caused faulty occlusion. During this inquiry, all orthodontic interventions were clinically successful for all participants. Thus, no participant was excluded after receiving the Invisalign trays and finishing the treatment. Also, no participant was lost for the follow up and all participants who were recruited into this study stayed till the study was finished and all assessments were carried out.

After the success of the Invisalign orthodontic treatment was ensured, each participant was requested to score the NEO-FFI and OHIP measures again following the same procedures before.

The main study outcome measures for this study were oral health impacts of Invisalign orthodontic treatment and personality characteristics.

During this investigation, intra-examiner reliability was tested by repeating 10 clinical evaluations by the same investigator (A.A.Al.) (Kappa value was 0.92), meanwhile inter-examiner reliability was tested by repeating the 10 clinical evaluations by another examiner (S.A.) (Kappa value was 0.88). This refers to that the assessment procedures were adequately reliable and repeatable. This study was reported following the STROBE guidelines.

### Statistical analysis

The statistical analysis for this inquiry was conducted utilizing the SPSS computer software (IBM SPSS Statistics v19.0; IBM Corp., USA). Descriptive statistics of various variables in this study were carried out and tabulated. Normal distribution of the data was tested using the Kolmogorov–Smirnov test. Dental and demographic variables variations between genders were assessed using Chi square and Fisher’s Exact tests. OHIP and NEO-FFI scores variations between genders as well as between different groups based on each dental and demographic variable were assessed using ANOVA test. For each gender and the total sample, within group comparisons of baseline and after treatment OHIP and NEO-FFI scores were performed via paired samples *t*-test. Correlations between OHIP scores and dental and demographic variables were assessed using Fisher’s Exact test. Associations between personality attributes and oral health impacts of Invisalign orthodontic treatment were initially assessed using the Pearson Correlation test to identify raw statistics before considering the confounding effects of demographic and dental variables. Hierarchical regression analysis (including dental and demographic variables in the first block and NEO-FFI scores in the second block) was used to predict OHIP scores and identify the contribution of NEO-FFI scores towards OHIP scores taking into consideration the confounding effects of demographic and dental factors on the association between OHIP and NEO-FFI scores. Significant outcomes were identified at two tailed α = 0.05 and a 95% confidence interval.

Confounding effects of demographic factors (age, gender, level of education, marital status, and smoking) and dental factors (incisor classification, canine classification, molar classification, skeletal classification, degree of overbite, degree of overjet, presence of cross bite, presence of scissor bite, the need for extraction, and presence of tooth crowding/spacing) were considered during the statistical analysis in this study. Stratification of data analysis by gender was performed to identify variations as well as associations between OHIP and NEO-FFI scores before and after treatment to avoid the confounding effects of gender. Also, hierarchical regression analysis (including dental and demographic variables in the first block and NEO-FFI scores in the second block) was conducted to take into consideration the confounding effects of dental and demographic variables on the contribution of NEO-FFI scores towards OHIP scores and the association between OHIP and NEO-FFI scores; i.e. the ability of NEO-FFI scores to predict OHIP scores.

For this inquiry, a computer software (G*Power, version 3.1.9.7; Heinrich-Heine University) was employed for sample size estimation. An effect size of 0.333 was calculated considering an R^2^ of 0.25 for an a-priori power analysis utilizing F test for fixed model multiple regression analysis in the software. A sample size of 45 participants was determined via the software to meet requirements for statistic power (1 − β) of 0.8, effect size of 0.333, and significance level (α) of 0.05. Extra numbers of participants were allowed to join this inquiry to equilibrate for any potential dropouts or exclusions. None of the participants were excluded after inclusion and none of the participants were lost to follow up (drop out ratio is 0%).

## Results

In total, 50 participants (24 males and 26 females) who received Invisalign treatment were recruited into the study and had their data analyzed. Participants’ age ranged between 18 and 48 years old (mean age = 27.62 ± 8.25 years, SE = 1.17, 95% CI = 24.71–29.89). Females’ age ranged between 18 and 45 years old (mean age = 27.42 ± 7.69 years, SE = 1.51, 95% CI = 24.60–30.59); meanwhile, males’ age ranged between 17 and 48 years old (mean age = 27.92 ± 8.87 years, SE = 1.81, 95% CI = 24.17–32.18).

Table 1 presents gender differences and distribution of frequencies, percentages, standard errors, and 95% confidence intervals of different demographic and dental variables among the study participants. Females reported more smoking than males (X^2^ = 13.542, *P* < 0.001). Females had more Class I, less Class II and less Class III incisor relationships than males (Fisher’s Exact Value = 7.671, *P* = 0.022). Also, females had more Class I, less Class II, and less Class III skeletal relationships than males (Fisher’s Exact Value = 6.520, *P* = 0.039). In addition, females had higher frequency of normal overjet and less frequency of increased overjet than males (X^2^ = 7.964, *P* = 0.019).

**Table 1:** Gender differences and distribution of frequencies, percentages, standard errors, and 95% confidence intervals of demographic and dental variables among the study participants (n = 50, 24 males and 26 females).

Variable	Male				Female				Statistic value (*P*)
	frequency (%)	SE	95% CI		frequency (%)	SE	95% CI		
			Lower	Upper			Lower	Upper	
*Education*									
High school	7 (29.2)	9.2	9.7	52.8	5 (19.2)	8.8	2.6	42.3	.810^#^ (.680)
Bachelor	15 (62.5)	8.4	45.8	77.8	18 (69.2)	10.0	50.0	88.5	
PhD	2 (8.3)	5.5	.0	23.6	3 (11.5)	6.3	.0	25.6	
*Marital status*									
Single	17 (70.8)	9.5	51.4	91.7	20 (76.9)	9.6	51.3	91.0	.241^$^ (.624)
Married	7 (29.2)	9.5	8.3	48.6	6 (23.1)	9.6	9.0	48.7	
*Smoking*									
No	10 (41.7)	10.7	16.7	69.4	0 (0%)	–	–	–	13.542^#^ (< .001)
Yes	14 (58.3)	10.7	30.6	83.3	26(100.0)	.0	100.0	100.0	
*Incisor class*									
Class I	8 (33.3)	8.4	9.7	48.6	18 (69.2)	9.6	47.5	84.6	7.671^#^ (.022)
Class II	10 (41.7)	9.7	25.0	62.5	7 (26.9)	8.4	12.8	44.9	
Class III	6 (25.0)	8.0	9.7	44.4	1 (3.8)	3.9	.0	14.1	
*Canine class*									
Class I	10 (41.7)	10.1	20.8	62.4	18 (69.2)	9.6	47.5	84.6	4.350^#^ (.111)
Class II	10 (41.7)	9.7	25.0	62.5	7 (26.9)	8.4	12.8	44.9	
Class III	4 (16.7)	6.8	5.6	33.3	1 (3.8)	3.9	.0	14.1	
*Molar class*									
Class I	10 (41.7)	10.1	20.8	62.4	18 (69.2)	9.6	47.5	84.6	4.350^#^ (.111)
Class II	10 (41.7)	9.7	25.0	62.5	7 (26.9)	8.4	12.8	44.9	
Class III	4 (16.7)	6.8	5.6	33.3	1 (3.8)	3.9	.0	14.1	
*Skeletal class*									
Class I	11 (45.8)	10.5	22.2	69.4	21 (80.8)	8.1	56.4	92.3	6.520^#^ (.039)
Class II	9 (37.5)	9.5	20.8	61.1	4 (15.4)	6.7	7.7	30.8	
Class III	4 (16.7)	6.8	5.6	33.3	1 (3.8)	3.9	.0	14.1	
*Over bite*									
Deep bite	8 (33.3)	9.1	13.9	52.8	8 (30.8)	8.5	15.4	50.0	5.929^$^ (.052)
Normal	4 (16.7)	7.6	4.2	33.3	12 (46.2)	7.6	29.5	62.8	
Open bite	12 (50.0)	10.3	33.3	73.6	6 (23.1)	7.5	9.0	41.0	
*Over jet*									
Decreased	9 (37.5)	9.1	20.8	58.3	9 (34.6)	9.1	14.1	48.7	7.964^$^ (.019)
Normal	4 (16.7)	7.0	4.2	29.2	13 (50.0)	9.5	28.2	71.8	
Increased	11 (45.8)	10.1	29.2	66.7	4 (15.4)	6.4	5.1	30.8	
*Cross bite*									
No	4 (16.7)	7.0	4.2	31.9	6 (23.1)	7.9	9.0	44.9	.321^#^ (.728)
Yes	20 (83.3)	7.0	68.1	95.8	20 (76.9)	7.9	55.1	91.0	
*Scissor bite*									
No	3 (12.5)	6.4	1.4	27.8	1 (3.8)	4.0	.0	15.4	1.270^#^ (.340)
Yes	21 (87.5)	6.4	72.2	98.6	25 (96.2)	4.0	84.6	100.0	
*Extraction*									
No	8 (33.3)	10.1	18.1	61.1	12 (46.2)	8.5	30.8	64.1	.855^$^ (.355)
Yes	16 (66.7)	10.1	38.9	81.9	14 (53.8)	8.5	35.9	69.2	
*Crowding and spacing*									
Crowding present	14 (58.3)	12.4	29.2	86.1	14 (53.8)	9.3	39.8	80.8	.830^$^ (.660)
Normal	6 (25.0)	10.4	8.3	51.4	5 (19.2)	7.5	3.8	34.6	
Spacing present	4 (16.7)	6.9	4.2	29.2	7 (26.9)	8.2	11.5	44.9	

SE = Standard error, CI = 95% confidence intervals of the percent, $ = Calculated via Chi Square test, # = Calculated via Fisher’s Exact test.

Table 2 presents gender differences and distribution of means, standard deviation, standard errors, and 95% confidence intervals of age, OHIP scores before and after treatment, and NEO-FFI scores before and after treatment among the study participants. None of these variables were different between genders (*P* > 0.05) (Table 2).

**Table 2:** Gender differences and distribution of means, standard deviation, standard errors, and 95% confidence intervals of age, OHIP scores before and after treatment, and NEO-FFI scores before and after treatment among the study participants (n = 50, 24 males and 26 females).

Variable	Male					Female					F	P
	Mean	SD	SE	95% CI		Mean	SD	SE	95% CI			
				Lower	Upper				Lower	Upper		
Age	27.92	8.866	1.90	24.17	32.18	27.42	7.685	1.33	24.60	30.59	.044	.834
OHIP Before	10.88	6.873	1.15	9.11	13.18	14.15	7.052	1.41	11.13	17.34	2.764	.103
OHIP After	12.25	9.275	1.96	8.95	17.32	8.12	4.803	.88	6.63	10.33	4.008	.051
N Before	20.92	6.965	1.49	17.81	24.24	20.27	6.403	1.51	16.90	23.36	.117	.733
N After	21.29	5.782	1.13	18.43	23.35	20.92	7.652	1.55	16.44	23.36	.036	.849
E Before	29.00	4.578	.95	27.29	30.97	27.00	4.891	.91	25.07	29.09	2.219	.143
E After	28.92	4.680	.92	26.94	30.71	27.96	4.762	1.05	26.35	30.36	.510	.478
O Before	23.54	4.293	.78	21.97	25.21	23.00	6.493	1.18	20.26	25.06	.119	.732
O After	22.00	3.934	.63	20.56	23.13	23.38	5.185	1.02	20.21	25.11	1.117	.296
A Before	26.29	3.759	.78	24.72	28.44	27.54	4.641	.84	25.47	29.92	1.079	.304
A After	26.58	4.106	.83	25.17	28.68	26.31	4.343	.72	24.72	27.60	.053	.819
C Before	34.92	5.469	1.06	32.53	37.09	34.81	5.485	1.01	32.57	36.76	.005	.944
C After	33.88	6.031	1.16	31.63	36.21	34.08	5.628	1.08	31.33	36.71	.015	.903

SD = Standard deviation, SE = Standard error of the mean, CI = 95% confidence intervals of the mean, OHIP = Oral Health Impact Profile score, Before = Before treatment, After = After treatment, N = Neuroticism, E = Extraversion, O = Openness, A = Agreeableness, C = Conscientiousness, F = F Statistic value using ANOVA test to compare males and females groups (degree of freedom = 1 between groups and 48 within groups), *P* = Probability value.

### Comparison of OHIP and NEO-FFI scores before and after treatment

For each gender, within group comparisons of baseline and after treatment OHIP and NEO-FFI scores were performed via paired samples *t*-test. No differences between baseline OHIP and NEO-FFI scores and after treatment OHIP and NEO-FFI scores were found among males as well as among females except that females scored less OHIP scores after treatment (had less negative impacts) in comparison to baseline OHIP scores (t = 3.782, df = 25, *P* = 0.001, 95% CI of mean difference = 2.750–9.327) (Table 3).

**Table 3:** Comparison of OHIP and NEO-FFI scores before and after treatment among the study sample (n = 50, 26 females and 24 males).

Group	Variable Pairs	Mean difference	SE Mean	95% CI of difference		t	df	P
				Lower	Upper			
All Sample	OHIP Before–OHIP After	2.480	1.386	− .305	5.265	1.789	49	.080
	N Before–N After	− .520	.902	− 2.332	1.292	− .577	49	.567
	E Before–E After	− .460	.844	− 2.156	1.236	− .545	49	.588
	O Before–O After	.540	.802	− 1.071	2.151	.674	49	.504
	A Before–A After	.500	.726	− .958	1.958	.689	49	.494
	C Before–C After	.880	.987	− 1.104	2.864	.891	49	.377
Males	OHIP Before–OHIP After	− 1.375	2.069	− 5.656	2.906	− .664	23	.513
	N Before–N After	− .375	.961	− 2.363	1.613	− .390	23	.700
	E Before–E After	.083	1.111	− 2.216	2.382	.075	23	.941
	O Before–O After	1.542	1.025	− .579	3.662	1.504	23	.146
	A Before–A After	− .292	.829	− 2.006	1.422	− .352	23	.728
	C Before–C After	1.042	1.337	− 1.724	3.808	.779	23	.444
Females	OHIP Before–OHIP After	6.038	1.597	2.750	9.327	3.782	25	.001
	N Before–N After	− .654	1.510	− 3.764	2.456	− .433	25	.669
	E Before–E After	− .962	1.270	− 3.578	1.655	− .757	25	.456
	O Before–O After	− .385	1.208	− 2.872	2.103	− .318	25	.753
	A Before–A After	1.231	1.165	− 1.169	3.631	1.056	25	.301
	C Before–C After	.731	1.467	− 2.291	3.752	.498	25	.623

SE Mean = Standard error mean, CI = Confidence intervals, t = t statistics, df = Degree of freedom, *P* = Significance probability value, OHIP = Oral Health Impact Profile score, Before = Before treatment, After = After treatment, N = Neuroticism, E = Extraversion, O = Openness, A = Agreeableness, C = Conscientiousness.

### Comparison of OHIP and NEO-FFI scores between groups according to demographic variables

ANOVA test (followed by LSD Post hoc test where the number of compared groups or variable categories was above two) revealed that OHIP and NEO-FFI scores before and after treatment were not significantly different between different levels of education, being married or single, being smoker or not, different canine classifications, different molar classifications, different skeletal classifications, degree of overbite, having cross bite or not, having scissor bite or not, and being treated with extraction or not (*P* > 0.05).

On the other hand, participants with class III incisor relationship reported higher OHIP scores after treatment than those with class I incisor relationship (LSD Post hoc test: Mean difference = 6.681, SE = 3.116, *P* = 0.037, CI = 0.41–12.95), although ANOVA showed no significant differences between different incisor classifications (ANOVA: F = 2.342, df = 2, *P* = 0.107) . Also, the degree of overjet showed differences according to OHIP scores after treatment (ANOVA: F = 3.195, df = 2, *P* = 0.050); LSD Post hoc test showed that increased overjet was associated with higher OHIP scores (i.e. associated with worse oral health impacts) than normal overjet before treatment (Mean difference = 6.376, SE = 2.551, *P* = 0.016, CI = 1.24–11.51). In addition, presence of spacing was associated with higher agreeableness scores after treatment (LSD Post hoc test: Mean difference = 4.091, SE = 1.721, *P* = 0.022, CI = 0.63–7.55), although ANOVA showed no significant differences between different crowding/spacing conditions (spacing, no spacing and crowding) (ANOVA: F = 2.894, df = 2, *P* = 0.065). Participants with spacing also demonstrated higher conscientiousness scores after treatment than those with crowding (mean difference = 4.364, SE = 1.988, *P* = 0.033, CI = 0.36–8.36), although ANOVA showed no significant differences between different crowding/spacing conditions (spacing, no spacing and crowding) (ANOVA: F = 2.587, df = 2, *P* = 0.086).

### Correlations between variables before considering the effects of confounding variables

Before as well as after treatment, no significant correlations (*P* > 0.05) were found between OHIP scores and NEO-FFI scores (Table 4). Similarly, no significant relations were found between OHIP scores and each of gender, different levels of education, being married or single, being smoker or not, different incisor classifications, different canine classifications, different molar classifications, different skeletal classifications, degree of overbite, degree of overjet, having cross bite or not, having scissor bite or not, being treated with extraction or not, and having crowding/spacing (*P* > 0.05) except that people with reversed overjet scored higher OHIP scores than participants with normal overjet (Fisher’s Exact test value = 47.491, *P* = 0.016) (Table 5).

**Table 4:** Correlations between OHIP and NEO-FFI scores before and after treatment among the study population (n = 50, 26 females and 24 males).

Correlated variables		Total sample	Females	Males
OHIP and N before Tt	R	− .155	− .022	− .277
	P	.282	.914	.190
OHIP and E before Tt	R	.020	.271	− .163
	P	.888	.180	.447
OHIP and O before Tt	R	.143	.178	.134
	P	.323	.384	.534
OHIP and A before Tt	R	− .036	− .232	.145
	P	.803	.253	.500
OHIP and C before Tt	R	.084	.134	.039
	P	.560	.513	.856
OHIP and N after Tt	R	.212	.132	.314
	P	.139	.521	.135
OHIP and E after Tt	R	.209	.215	.189
	P	.146	.291	.377
OHIP and O after Tt	R	.020	− .032	.150
	P	.890	.875	.484
OHIP and A after Tt	R	− .004	.060	− .059
	P	.978	.772	.785
OHIP and C after Tt	R	.085	.134	.078
	P	.558	.513	.719

OHIP = Oral Health Impact Profile scores, Before Tt = Before treatment, After Tt = After treatment, N = Neuroticism, E = Extraversion, O = Openness, A = Agreeableness, C = Conscientiousness, R = Pearson’s Correlation Coefficient, *P* = Two tailed probability value.

**Table 5:** Correlations between OHIP scores and different demographic and dental variables before and after treatment among the study population (n = 50, 26 females and 24 males).

Correlated variables		Total sample (n = 50)		Females (n = 26)		Males (n = 24)	
		Before Tt	After Tt	Before Tt	After Tt	Before Tt	After Tt
OHIP and gender	FE	19.538	21.888	–	–	–	–
	P	.596	.309	–	–	–	–
OHIP and education	FE	43.239	37.951	28.015	28.826	36.348	27.347
	P	.305	.904	.613	.640	.383	.985
OHIP and marital status	FE	24.293	23.059	15.097	14.404	16.653	16.076
	P	.087	.180	.277	.428	.377	.153
OHIP and smoking	FE	18.673	24.899	$	$	13.938	16.459
	P	.672	.067	$	$	.927	.126
OHIP and incisor class	FE	40.819	38.198	34.218	34.218	27.810	25.433
	P	.393	.755	.846	.896	.982	.797
OHIP and canine class	FE	43.320	37.927	34.218	34.218	28.530	25.788
	P	.319	.916	.846	.896	.991	.861
OHIP and molar class	FE	43.320	37.927	34.218	34.218	28.530	25.788
	P	.319	.916	.846	.896	.991	.861
OHIP and skeletal class	FE	42.105	37.523	34.817	36.778	28.500	25.738
	P	.442	.932	.866	.670	.992	.867
OHIP and overbite	FE	33.384	40.938	27.053	24.045	27.031	27.791
	P	.953	.292	.428	.943	1.000	.425
OHIP and overjet	FE	33.345	47.491	23.655	31.397	28.500	27.935
	P	.955	.016	.985	.075	.992	.391
OHIP and cross bite	FE	18.673	20.658	12.900	15.791	16.850	13.857
	P	.672	.438	.666	.227	.466	.648
OHIP and scissor bite	FE	24.895	23.743	23.850	21.771	17.356	13.885
	P	.258	.574	.308	1.000	.640	.853
OHIP and extraction	FE	20.300	17.233	13.701	10.575	14.796	11.593
	P	.475	.841	.493	1.000	.756	.878
OHIP and crowding/spacing	FE	42.245	35.125	28.038	24.454	32.355	25.106
	P	.185	.906	.331	.914	.410	.942

OHIP = Oral Health Impact Profile scores, Before Tt = Before treatment, After Tt = After treatment, FE = Fisher’s Exact test value, *P* = Two tailed probability value. $ = Not computed because it is constant.

### Regression analysis and prediction of OHIP scores using NEO-FFI scores

Considering the effects of confounding variables, hierarchical regression analysis (including NEO-FFI scores in the second block and all other variables in the first block) to predict OHIP scores before treatment showed that overjet (R^2^ = 0.505, B = − 4.658, 95% CI for B = − 8.515–− 0.801, t = − 2.467, *P* = 0.020) and crossbite (R^2^ = 0.505, B = − 10.816, 95% CI for B = − 18.766–− 2.866, t = − 2.779, *P* = 0.009) were useful to predict OHIP scores before treatment; meanwhile, NEO-FFI scores before treatment were not useful to predict OHIP scores before treatment (*P* > 0.05). On the other hand, demographic factors as well as NEO-FFI scores after treatment were not useful to predict OHIP scores after treatment (*P* > 0.05).

In order to control for the confounding effects of gender, the data was split according to gender and the regression analysis was conducted for each gender alone.

Among males before treatment (Table 6), hierarchical regression analysis showed that marital status, presence of crowding and spacing, and openness scores before treatment were able to predict OHIP scores before treatment (R^2^ = 0.911, *P* = 0.048); meanwhile, other NEO-FFI scores before treatment were not useful to predict OHIP scores before treatment (*P* > 0.05). Among females, however, demographic factors as well as NEO-FFI scores before treatment were not useful to predict OHIP scores before treatment (*P* > 0.05).

**Table 6:** Hierarchical regression analysis using NEO-FFI scores and other variables in to predict OHIP scores before and after treatment among male participants in the study (n = 24 males).

Dependent variable	Predictors	Unstand Co		Stand Co	t	Sig	95% CI for B	
		B	Std. error	Beta			Lower bound	Upper bound
OHIP before R^2^ = .911	(Constant)	− 15.102	17.106	–	− .883	.418	− 59.074	28.870
	Age	− 1.000	.453	− 1.290	− 2.210	.078	− 2.164	.163
	Education level	7.643	4.442	.887	1.721	.146	− 3.775	19.060
	Marital status	21.453	7.673	1.449	2.796	.038	1.730	41.177
	Smoking	7.288	3.261	.534	2.235	.076	− 1.095	15.672
	Incisor class	− 4.034	3.592	− .455	− 1.123	.312	− 13.268	5.200
	Molar class	4.265	9.073	.457	.470	.658	− 19.059	27.588
	Skeletal class	− 4.135	9.413	− .452	− .439	.679	− 28.332	20.061
	Overbite	− 3.781	3.173	− .504	− 1.192	.287	− 11.937	4.375
	Overjet	3.947	3.554	.533	1.111	.317	− 5.190	13.085
	Cross bite	4.094	11.004	.227	.372	.725	− 24.193	32.380
	Scissor bite	− 10.207	12.647	− .502	− .807	.456	− 42.717	22.304
	Extraction	4.629	3.039	.324	1.523	.188	− 3.184	12.442
	Crowding/Spacing	6.347	2.445	.716	2.595	.049	.060	12.633
	N Before	− 3.667	2.107	− .521	− 1.740	.142	− 9.083	1.750
	E Before	4.053	2.895	.468	1.400	.220	− 3.390	11.496
	O Before	5.235	2.012	.632	2.601	.048	.062	10.407
	A Before	− 5.772	4.198	− .630	− 1.375	.228	− 16.563	5.018
	C Before	− .112	2.125	− .014	− .053	.960	− 5.574	5.349
OHIP after R^2^ = .959	(Constant)	− 84.641	17.105		− 4.948	.004	− 128.612	− 40.671
	Age	.119	.257	.113	.461	.664	− .543	.780
	Education level	9.087	2.624	.781	3.464	.018	2.343	15.832
	Marital status	− 27.299	6.649	− 1.367	− 4.106	.009	− 44.391	− 10.207
	Smoking	7.145	2.777	.388	2.573	.050	.007	14.284
	Incisor class	19.584	3.505	1.638	5.587	.003	10.573	28.594
	Molar class	37.958	9.481	3.017	4.003	.010	13.586	62.331
	Skeletal class	− 49.067	8.924	− 3.971	− 5.499	.003	− 72.007	− 26.128
	Overbite	14.202	3.806	1.404	3.732	.014	4.419	23.984
	Overjet	− 12.780	3.816	− 1.280	− 3.349	.020	− 22.589	− 2.971
	Cross bite	− 5.932	8.916	− .243	− .665	.535	− 28.851	16.987
	Scissor bite	47.545	10.914	1.732	4.356	.007	19.489	75.601
	Extraction	− 12.616	3.481	− .655	− 3.624	.015	− 21.565	− 3.668
	Crowding/spacing	− 8.813	2.203	− .737	− 4.001	.010	− 14.476	− 3.150
	N After	− 3.328	2.074	− .298	− 1.605	.169	− 8.658	2.003
	E After	− 8.224	2.482	− .761	− 3.313	.021	− 14.605	− 1.843
	O After	21.795	4.302	1.694	5.067	.004	10.737	32.853
	A After	− 3.771	1.807	− .315	− 2.087	.091	− 8.416	.874
	C After	10.293	2.138	1.008	4.813	.005	4.796	15.790

OHIP = Oral Health Impact Profile scores, R^2^ = Coefficient of determination, Before = Before treatment, After = After treatment, N = Neuroticism, E = Extraversion, O = Openness, A = Agreeableness, C = Conscientiousness, Unstand Co = Unstandardized coefficient, Stand Co = Standardized coefficient, B = Beta statistics, Std. Error = Standard Error, t = t statistics, Sig = Significance of probability (*P* value), CI = Confidence intervals. During the hierarchical multiple regression analysis, gender, age, education level, marital status, smoking, incisor class, canine class, molar class, skeletal class, overbite, overjet, cross bite, scissor bite, extraction, and crowding/spacing variables were included in the first block of independent variables and the neuroticism, extraversion, openness, agreeableness, and conscientiousness NEO-FFI dimension scores were included in the second block of independent variables to account for the confounding effects of first block variables on the contribution of NEO-FFI scores towards the OHIP scores (i.e. ability of NEO-FFI scores to predict OHIP scores).

Among males after treatment (Table 6), hierarchical regression analysis showed that education level, marital status, smoking, incisor classification, molar classification, skeletal classification, overbite, overjet, scissor bite, extraction, presence of crowding and spacing, extraversion scores after treatment, openness scores after treatment, and conscientiousness scores after treatment were useful to predict OHIP scores after treatment (R^2^ = 0.959, *P* < 0.05); meanwhile, other personality scores after treatment (neuroticism and agreeableness) were not useful to predict OHIP scores after treatment (*P* > 0.05). However among females, demographic factors as well as NEO-FFI scores after treatment were not useful to predict OHIP scores after treatment (*P* > 0.05).

## Discussion

This inquiry demonstrated that personality scores contributed to oral health impacts before and after Invisalign orthodontic treatment; therefore, the null hypothesis was rejected.

In this study, females scored lower OHIP scores after treatment in comparison to baseline; meanwhile, males had no differences between baseline and after treatment OHIP scores. This might be attributed to the effects of personality factors that were found to play a role in predicting and influencing OHIP scores among males but not females. This might have affected the response of males after treatment and lead to no difference from baseline. On the other hand, females had no such effects and thus were able to identify the treatment effects which could have improved their clinical status and reflected on their better oral health impacts without the presence of modifying effects of personality in contrast to males. This might also be attributed to that females are more considerate towards inadequacies in dental and oral health, and give higher attention to their dental and oral health^2,4,6^.

This contrasts with previous studies that reported no relation between gender and impacts and satisfaction with orthodontic treatment^2,7,9–11^. This variation could be due to differences in type of used orthodontic treatment, racial factors, psychosocial considerations, timing of assessment throughout the study, and study design.

However, the results of this study agrees with previous studies that reported females to be more keen to accept and be more satisfied with orthodontic therapy^31^, more welling to undergo orthodontic therapy^32^, and have less functional limitations due to orthodontic treatment than males^33^.

In addition, this is in line with the conclusions that orthodontic treatment is associated with improved oral health related quality of life^17^. However, this contrasts with the findings of other studies that reported no difference in attitudes towards orthodontic therapy^9–11^. This variation could be due to differences in treatments or racial and social backgrounds. Differences in patient compliance as well as expectations could also underlie this variation^34^.

Removable orthodontic treatment impacts daily living to lesser extent than fixed orthodontics^35^. In this context, previous studies reported positive changes and high satisfaction with Invisalign treatment especially with eating, chewing and appearance^19,36,37^. Invisalign treatment was associated with better satisfaction, better oral health related quality of life, and less negative impacts on oral health than fixed orthodontics^12–16,19,20,37,38^. They were associated with less disruption to eating and chewing and were rated as more satisfactory than fixed appliances^16^. Also, oral health related quality of life was not significantly affected by Invisalign treatment^38^. This could be attributed to that Invisalign was associated with good oral health and periodontal parameters as well as minimal pain, no halitosis, dry mouth or plaque accumulation^12–16,38^.

In addition, this could be explained by their superior appearance and being removable which have implications on psychosocial and functional aspects of individuals^16,18,19^.

Furthermore, Invisalign and clear aligners were associated with less pain than fixed orthodontics^13–16^. This might be explained by that fixed orthodontic appliances could cause more tension, pressure, pain and sensitivity of teeth due to continuous force exerted by the appliance components. On the other hand, removable appliances exert intermittent force that permit the tissues to rest and reorganize before resuming the compressive forces again^16^.

Levels of pain due to orthodontic treatment are affected by individuals’ emotions, environment, psychology and psychosocial considerations^39^. This would have implications on oral health impacts following orthodontic treatment. Enhancement of dentofacial aesthetics and oral function are also linked to improvements in sociopsychological attributes of individuals^40^.

Previous studies reported higher satisfaction and more positive impacts after orthodontic treatment. This could be explained by the improved dental esthetics and function that result from treatment^2,7,9^. Non extraction treatment was associated with less satisfaction and more discomfort^2^. Also, higher orthodontic treatment needs were associated with more negative impacts on oral health related quality of life^41^.

In this study, extraversion, openness, and conscientiousness personality factors (NEO-FFI scores) were found to contribute to oral health related quality of life (OHIP scores). In this regard, extraversion could be associated with more enthusiasm to identify any changes to oral situation and oral health related impacts. Also, openness could be associated with more readiness to demonstrate opinions, worries, and attitudes towards changes to oral situation and oral health related impacts. In addition, conscientiousness could be associated with more commitment, organization, and reporting changes in oral status and oral health related impacts.

This agrees with the findings of previous studies that linked personality traits with oral health related quality of life that associated other types of orthodontic treatment^2,41–46^. Higher scores of extraversion and openness personality traits were accompanied with less impact of orthodontic treatment needs on oral health related quality of life^42^. Also, less satisfaction and more negative impacts after orthodontic treatment were associated with higher levels of neuroticism^2^. In addition, extraversion and neuroticism profiles were associated with oral health related quality of life^41^. However, the relation between orthodontic treatment needs and oral health related quality of life was not found to be mediated by personality factors^41^. Moreover, extraversion was associated with facial attractiveness while neuroticism was associated with smile attractiveness^43^. Personality traits also affect willingness and compliance with orthodontic treatment^44,45^, and patient cooperation with early orthodontic treatment^46^. Furthermore, previous studies reported reduced neuroticism and increased openness, agreeableness and conscientiousness scores after fixed orthodontic treatment^7^.

However, this contrasts with the findings of previous studies that found no association between personality and each of attitudes towards orthodontic treatment^9^, perception of pain after orthodontic treatment^9^, compliance with treatment^10^, and cooperation during orthodontic treatment^11^. Moreover, lower extraversion scores were found to be associated with more cooperation with orthodontic treatment^11^.

This variation could be attributed to variations in racial backgrounds, cultural factors, social factors, differences in applied treatments, study designs, timing of personality assessment throughout the study, assessment tools used to evaluate personality, and demographic factors. This variation could also be due to that some studies were cross sectional investigations that controlled the treated patients with groups of non treated individuals^9^. Meanwhile, the present inquiry is a within subject cross over longitudinal study that compared the same participants at baseline and after treatment. The within subject cross over longitudinal design of this study allowed avoidance of interindividual variations and provided standardized baseline for comparison of the effects of treatment meanwhile reduces the required numbers of participants because each participant will serve as a control for him/her self, so the participants’ baseline status would be the control for after treatment within the same participant^38^.

Up to the authors’ knowledge, the outcomes of this investigation are the first within subject assessment into the association between personality profiles and oral health related quality of life among patients treated with Invisalign orthodontic treatment. Also, the assessment was conducted following standardized and controlled settings utilizing comprehensive, reliable, and valid measures to explore this association.

The above results and discussion show that Invisalign orthodontic treatment has impacts on oral health related quality of life among females. It has links to personality factors and attributes among males. Therefore, it is worth considering these factors when treatment with Invisalign is planned in order to achieve best treatment outcomes and meet patients’ expectations. This would potentially enhance treatment acceptance and patients’ compliance with treatment. Assessment of personality factors, appraisal of oral health related quality of life, patient education, and assessment of patients’ expectations could potentially contribute to treatment success in this regard.

Study limitations include the potential effects of racial, cultural, religious, economic, and social factors on the association between oral health related quality of life and personality attributes and factors. However in this study, confounding effects of many factors including age, gender, level of education, marital status, smoking and dental factors were accounted for and considered in the analysis during this research. Further investigations are required to assess the potential effects of racial, cultural, religious, social and economic factors on the association between oral health related quality of life and personality attributes and factors. Future research is also required to compare the association between oral health related quality of life and personality attributes and factors among different orthodontic treatments against Invisalign orthodontic treatment. In addition, further research is required among different populations utilizing larger study sample size.

## Conclusions

Within the limitations of this study, Invisalign orthodontic treatment was accompanied with less negative oral health impacts in comparison to baseline among females but not among males. Contribution of personality profiles towards the impacts of Invisalign treatment on oral health related quality of life is different between genders. Personality attributes and factors; openness before treatment and extraversion, openness, and conscientiousness after treatment, were linked to and able to predict oral health impacts of Invisalign orthodontic treatment among males.

## Data Availability

Data generated and analysed during this study are available from the corresponding author upon request to the following email: alomirim@yahoo.co.uk.
